# Supplementary material for: Machine learning for differentiating lung squamous cell cancer from adenocarcinoma using Clinical-Metabolic characteristics and 18F-FDG PET/CT radiomics
Source: PLoS One. 2024 Apr 3;19(4):e0300170. doi: 10.1371/journal.pone.0300170 (PMC10990193; doi:10.1371/journal.pone.0300170)
Supplement: S1 Table — (DOC) [file pone.0300170.s001.doc]

**S1 Table Radiomics feature extraction**

All handcrafted features were obtained using a custom feature analysis program implemented in Pyradiomic (http://pyradiomics.readthedocs.io).

| Categories | Subcategories | Features | Number |
| --- | --- | --- | --- |
| Shape-based |  | Maximum 3D Diameter, Maximum 2D Diameter Slice, Sphericity, Minor Axis, Elongation, Surface Volume Ratio, Volume, Major Axis, Surface Area, Flatness, Least Axis, Maximum 2D Diameter Column, Maximum 2D Diameter Row | 13 |
| First-order histogram |  | Interquartile Range, Skewness, Uniformity, Median, Energy, Robust Mean Absolute Deviation, Mean Absolute Deviation, Total Energy, Maximum, Root Mean Squared, 90Percentile, Minimum, Entropy, Range, Variance, 10Percentile, Kurtosis, Mean | 18 |
| Texture | Gray Level Dependence Matrix (GLDM) | Gray Level Variance, High Gray Level Emphasis, Dependence Entropy, Dependence Non-Uniformity, Gray Level Non-Uniformity, Small Dependence Emphasis, Small Dependence High Gray Level Emphasis, Dependence Non-Uniformity Normalized, Large Dependence Emphasis, Large Dependence Low Gray Level Emphasis, Dependence Variance, Large Dependence High Gray Level Emphasis, Small Dependence Low Gray Level Emphasis, Low Gray Level Emphasis | 14 |
|  | Gray Level Co-occurrence Matrix (GLCM) | Joint Average, Contrast, Joint Entropy, Cluster Shade, Maximum Probability, Idmn, Joint Energy, Difference Entropy, Inverse Variance, Difference Variance, Idn, Idm, Correlation, Autocorrelation, Sum Entropy, Sum Squares, Cluster Prominence, Imc2, Imc1, Difference Average, Id, Cluster Tendency | 22 |
|  | Gray Level Run Length Matrix (GLRLM) | Short Run Low Gray Level Emphasis, Gray Level Variance, Low Gray Level Run Emphasis, Gray Level Non-Uniformity Normalized, Run Variance, Gray Level Non-Uniformity, Long Run Emphasis, Short Run High Gray Level Emphasis, Run Length Non-Uniformity, Short Run Emphasis, Long Run High Gray Level Emphasis, Run Percentage, Long Run Low Gray Level Emphasis, Run Entropy, High Gray Level Run Emphasis, Run Length Non-Uniformity Normalized | 16 |
|  | Gray Level Size Zone Matrix (GLSZM) | Gray Level Variance, Zone Variance, Gray Level Non-Uniformity Normalized, Size Zone Non-Uniformity Normalized, Size Zone Non-Uniformity, Gray Level Non-Uniformity, Large Area Emphasis, Small Area High Gray Level Emphasis, Zone Percentage, Large Area Low Gray Level Emphasis, Large Area High Gray Level Emphasis, High Gray Level Zone Emphasis, Small Area Emphasis, Low Gray Level Zone Emphasis, Zone Entropy, Small Area Low Gray Level Emphasis | 16 |
|  | Neighbouring Gray Tone Difference Matrix (NGTDM) | Coarseness, Complexity, Strength, Contrast, Busyness | 5 |
