# Supplementary material for: Machine learning for differentiating lung squamous cell cancer from adenocarcinoma using Clinical-Metabolic characteristics and 18F-FDG PET/CT radiomics
Source: PLoS One. 2024 Apr 3;19(4):e0300170. doi: 10.1371/journal.pone.0300170 (PMC10990193; doi:10.1371/journal.pone.0300170)
Supplement: S1 File — (DOCX) [file pone.0300170.s004.docx]

**Approval Form for Ethical Review of the Affiliated Cancer Hospital of**

**Shandong First Medical University**

| **1. Number（To be completed by the Ethics Committee）: SDTHECX 2023010008** | |
| --- | --- |
| **2. Research Title：Machine Learning for Differentiating Lung Squamous Cell Cancer From Adenocarcinoma Using Clinical-metabolic Characteristics and 18F-FDG PET/CT Radiomics** | |
| **3. Research Leader** | |
| **Name:Yong Ying** | **Institution: Affiliated Cancer Hospital of**  **Shandong First Medical University** |
| **Title: Researcher** | **Email：****[yinyongsd@126.com](mailto:yinyongsd@126.com)** |
| **4. Research Program Overview**  1. Patients with inoperable non-small cell lung cancer(NSCLC), who were are diagnosed at stage III, were retrospectively analyzed.They were randomly divided into training set and validation set according to the ratio of 7:3.  2. 255 patients were randomly divided into training (n=177) and internal validation (n=78) cohorts.Clinical features were selected from the training cohort using univariate and multivariate Cox proportional hazards models; radiomic features were extracted from PET and CT images and fi1tered using least absolute shrinkage and selection operator and Cox proportional hazard regression.Three prediction models and a nomogram were then constructed using the previously selected clinical, CT and PET radiomics features.The predictive performance of the constructed models was evaluated using receiver operator characteristic curves, Kaplan Meier curves, and a nomogram.  3. To develop and validate a clinico-metabolic features and 18F-f1uorodeoxyglucose (FDG)positron emission tomography/computed tomography (PET/CT) radiomic-based nomogram via machine learning for the pretreatment prediction of discriminating between adenocarcinoma (ADC) and squamous cell carcinoma (SCC) in non-smal1 cell lung cancer (NSCLC).Based on the prediction model, a reliable, non-invasive and practical method to discriminate the histological subtypes of NSCLC patients before treatment is explored, so that more targeted treatment can be selected earlier. | |
| **5. Potential Ethical Issues and Measures to Address Them**  This study is a retrospective study and does not involve subject benefits and risks and adverse effects. | |
| 1. **Research Leader Assurance**   I declare that the contents filled out are true and I will conduct the research in strict accordance with the relevant contents of the approval form. I also declare that I will strictly abide by the national laws and relevant regulations, while protecting the health, rights and privacy of the research subjects. | |
| **The following content is filled in by the Ethics Committee** | |
| **7. Review Expert Opinion**  After review, the research value of the project is high, the research design is reasonable, the consideration of potential ethical issues is adequate, the treatment measures taken are effective and appropriate, and it meets the ethical requirements and is approved to carry out the research. | |
| **8. Ethics Committee Opinion**  The hospital ethics committee conducted a careful review of the researcher's qualifications, research protocols and related materials.It was concluded that the study did not violate relevant regulations and principles of human biomedical ethics.This project is agreed to be carried out, and informed consent can be waived.  02/10/2023 | |
