# Supplementary material for: Machine learning for differentiating lung squamous cell cancer from adenocarcinoma using Clinical-Metabolic characteristics and 18F-FDG PET/CT radiomics
Source: PLoS One. 2024 Apr 3;19(4):e0300170. doi: 10.1371/journal.pone.0300170 (PMC10990193; doi:10.1371/journal.pone.0300170)
Supplement: S2 File — (PDF) [file pone.0300170.s005.pdf]

山东第一医科大学附属肿瘤医院伦理委员会
临床研究伦理审查意见

编号：SDTHEC2023010008

项目名称：基于临床及代谢特征的机器学习模型结合 18F-FDG PET/CT 的影像组学模型区分肺鳞癌和腺癌

Machine learning based on clinico-Metabolic features integrated 18F-FDG PET/CT radiomics for distinguishing squamous cell carcinoma from adenocarcinoma of lung

项目负责人：尹勇	职称：研究员
项目联系人：张亚琳	联系电话：17799257743

审查类型：

☐课题申报

☑ 科研项目开展　课题批准机构：研究者发起　项目编号：________

☐延续审查课题

审查资料：

☑ 申请表　☐研究方案　☐知情同意书　☑ 其他资料 知情豁免申请书

（包括：实验用品安全性资料、生产企业资质证明、实验用品提供者的资质证明）

研究内容摘要：

1. 对不可手术的 III 期非小细胞肺癌（NSCLC）患者进行了回顾性分析，按照 7:3 比例随机划分为训练集和验证集，所有患者均完善基线 ^{18}F-FDG PET/CT 检查。

2. 本研究于 2023 年 9 月 25 日开始，至 2023 年 10 月 25 日结束。回顾性收集 2017 年至 2022 年山东第一医科大学附属肿瘤医院收治的 255 例接受基线 ^{18}F-FDG PET/CT 检查以及活检的 III 期不可手术 NSCLC 患者。收集所有患者年龄、性别、治疗方案、肿瘤相关抗原等病例资料，记录患者的病理活检结果，采用 R version 3.4.0 和 SPSS 25.0 软件进行数据分析。基于 PET/CT 图像，分别提取 CT 和 PET 影像组学特征，并采用 LASSO Cox 降维筛选影像组学特征；单因素和多因素 Cox 回归分析筛选鉴别病理的临床特征。利用所筛选的 CT 和 PET 影像组学特征及临床特征以及 PET 代谢参数构建模型及列线图预测病

理鉴别能力，利用 ROC 曲线、校准曲线、DCA 分析评估其性能。

3. 通过开发一种新的 PET/CT 影像组学联合模型作为病理预测工具，以丰富并补充现有的诊疗建议。基于预测模型，患者诊断 NSCLC 时，因无法活检或者活检标本不满足检测条件时，即可辅助早期预测患者的病理类型，以便尽早选择更有针对性的治疗手段。

4. 本研究为回顾性研究，不涉及受试者受益及风险及不良反应。

1. Patients with inoperable non-small cell lung cancer(NSCLC), who were are diagnosed at stage III, were retrospectively analyzed. They were randomly divided into training set and validation set according to the ratio of 7:3.

2. 255 patients were randomly divided into training (n=177) and internal validation (n=78) cohorts. Clinical features were selected from the training cohort using univariate and multivariate Cox proportional hazards models; radiomic features were extracted from PET and CT images and filtered using least absolute shrinkage and selection operator and Cox proportional hazard regression. Three prediction models and a nomogram were then constructed using the previously selected clinical, CT and PET radiomics features. The predictive performance of the constructed models was evaluated using receiver operator characteristic curves, Kaplan Meier curves, and a nomogram.

3. To develop and validate a clinico-metabolic features and 18F-fluorodeoxyglucose (FDG) positron emission tomography/computed tomography (PET/CT) radiomic-based nomogram via machine learning for the pretherapy prediction of discriminating between adenocarcinoma (ADC) and squamous cell carcinoma (SCC) in non-small cell lung cancer (NSCLC). Based on the prediction model, a reliable, non-invasive and practical method to discriminate the histological subtypes of NSCLC patients before treatment is explored, so that more targeted treatment can be selected earlier.

保密要点： 保密患者的基本信息

伦理委员会审批意见：

医院伦理委员会对研究者资质、研究方案及相关资料进行了认真审核。经

审核，认为该项研究未违反人类生物医学伦理有关规定和原则，同意此项目开展，并免除知情同意书。

The hospital ethics committee conducted a careful review of the researcher's qualifications, research protocols and related materials. It was concluded that the study did not violate relevant regulations and principles of human biomedical ethics. This project is agreed to be carried out, and informed consent can be waived.

伦理委员会（章）

主任委员/副主任委员签字：

2023 年 10 月 2 日
