# Supplementary material for: Machine learning for differentiating lung squamous cell cancer from adenocarcinoma using Clinical-Metabolic characteristics and 18F-FDG PET/CT radiomics
Source: PLoS One. 2024 Apr 3;19(4):e0300170. doi: 10.1371/journal.pone.0300170 (PMC10990193; doi:10.1371/journal.pone.0300170)
Supplement: Attachment — Submitted filename: Response to Reviewers.docx [file pone.0300170.s006.docx]

**Response to reviewers**

We would like to thank the reviewers for their insightful and constructive feedback. We feel that the referees’ input has enabled us to produce a greatly improved manuscript, whose novelty and impact are now more readily apparent in the introduction and discussion. Based on the reviewers*’* comments, we have made extensive alterations to the structure, format, presentation, and analysis of our findings, with particular attention to adhering to the standards of academic English.

The reviewer specifically raised some concerns regarding the novelty and significance of our work. In response,we have reformatted the introduction to more clearlyaddress a broader swath of the novelty of our work within the appropriate context in both the introduction and discussion sections. In addition, the reviewers also expressed concern about several technical issues. In response, we have addressed all of the reviewer’s technical concerns with a substantial amount of new data, supported by additional detailed explanations. Below the comments of the reviewers are response point by point and the revisions are indicated.

Dear Reviewer #1:

On behalf of my co-authors, we thanks very much for giving us the second opportunity to revise our manuscript. We deeply appreciate editors and reviewers for their positive and constructive comments. We would like to make an explanation to reviewer 1 and hope it could be accepted.
**Question 1:** The abstract is detailed enough but not concise enough. What's more, it should not contain subtitles.

**Response:** Thank you for your valuable feedback regarding the abstract of our manuscript. We appreciate your point about the necessity for conciseness and the observation that subtitles should not be included within the abstract.In accordance with your suggestions, we have revised the abstract to make it more concise by eliminating unnecessary details and focusing on the key findings and significance of our study. We have also removed all subtitles to ensure the abstract adheres to the standard format and flows smoothly as a single, cohesive paragraph.We believe these revisions have improved the clarity and readability of the abstract while still providing a comprehensive overview of our research. The revised abstract now succinctly encapsulates the essence of our study without compromising on the essential information required for readers to grasp its importance. We hope that these changes adequately address your concerns, and we are open to any further suggestions you may have. The revised abstract is below:

Noninvasive differentiation between the squamous cell carcinoma (SCC) and adenocarcinoma (ADC) subtypes of non-small cell lung cancer (NSCLC) could benefit patients who are unsuitable for invasive diagnostic procedures. Therefore, this study evaluates the predictive performance of a PET/CT-based radiomics model. It aims to distinguish between the histological subtypes of lung adenocarcinoma and squamous cell carcinoma, employing four different machine learning techniques. A total of 255 Non-Small Cell Lung Cancer (NSCLC) patients were retrospectively analyzed and randomly divided into the training (n = 177) and validation (n = 78) sets, respectively. Radiomics features were extracted, and the Least Absolute Shrinkage and Selection Operator (LASSO) method was employed for feature selection. Subsequently, models were constructed using four distinct machine learning techniques, with the top-performing algorithm determined by evaluating metrics such as accuracy, sensitivity, specificity, and the area under the curve (AUC). The efficacy of the various models was appraised and compared using the DeLong test. A nomogram was developed based on the model with the best predictive efficiency and clinical utility, and it was validated using calibration curves. Results indicated that the logistic regression classifier had the best predictive power in the validation cohort of the radiomic model. The combined model (AUC 0.870) exhibited superior predictive power compared to the clinical model (AUC 0.848) and the radiomics model (AUC 0.774) . In this study, we discovered that the combined model, refined by the logistic regression classifier, exhibited the most effective performance in classifying the histological subtypes of NSCLC.

**Question 2:** Relevant research is not sufficiently introduced. Please provide a detailed introduction to the progress in building predictive models based on radiomics and clinical data. Relevant research progress on other diseases can also be introduced.
**Response:**

Thank you for your constructive feedback regarding the introduction of relevant research in our manuscript. In response to your comment, we have expanded the introduction to include a more detailed discussion of the advancements in predictive model development within radiomics. We have also incorporated a review of significant studies that utilize both radiomic features and clinical data to enhance the accuracy and applicability of predictive models. We have made the following changes to the introduction:

Relevant studies suggest that certain clinical characteristics can aid in differentiating the diagnosis of lung adenocarcinoma from lung squamous cell carcinoma. These include factors such as age, smoking history, tumor diameter, imaging signs, and microvascular density[7-9]. However, the sole reliance on clinical features for classifying pathological tissues may be influenced by the subjective judgment of the physicians or by the heterogeneity and quantity of the samples, potentially leading to variability in diagnostic outcomes.

As medical and information technologies advance, there is an exponential growth in the volume of medical data, especially in the production of medical imaging. These images harbor extensive latent details pertinent to human health. Yet, the manual examination and interpretation of such data are not only time-consuming but also prone to human bias. Leveraging the capabilities of machine learning can significantly alleviate these issues by extracting sophisticated features and minimizing subjectivity. Radiomics entails the quantitative retrieval of characteristics from conventional medical imaging. The development of predictive or diagnostic models through machine learning techniques allows for the collection of data that can be assimilated into clinical decision-making tools, consequently improving the accuracy of diagnoses or prognosis.[10,11]. Zhu et al.[12] extracted 485 features from manually delineated tumor regions in 129 NSCLC patients. The results demonstrated that the area under the curve (AUC) for the training and validation sets reached 0.905 and 0.893, respectively, indicating that the imaging features have substantial efficacy in differentiating between lung adenocarcinoma and squamous cell carcinoma. Bashir et al.[13] analyzed the effectiveness of a random forest model utilizing CT image radiomics features, CT semantic features, and combined features in distinguishing lung adenocarcinoma from squamous cell carcinoma. The findings revealed that the random forest model based on radiomics features could non-invasively analyze the histological subtypes of NSCLC with an AUC of 1.

18F-fluorodeoxyglucose (FDG) PET/CT, which combines anatomical and metabolic information, is crucial for identifying primary tumors, staging diseases, and assessing treatment success. Radiomics based on PET/CT shows potential in differentiating ADC from SCC. Yan et al’s study[14] developed separate models based on PET and CT, as well as a combined PET/CT model. Among these, the combined model exhibited superior performance in predicting ADC, SCC, and metastasis. Additional studies have also discovered that the inclusion of clinical characteristics, such as gender and smoking history, further enhanced the classification performance, achieving an area under the curve (AUC) of 0.859, which surpassed the performance of radiomics alone [15, 16].

**Question 3:** At the same time, the highlights and innovations of this article also need to be elaborated on in the Introduction.

**Response:** We are grateful for your recommendation, which has undoubtedly strengthened the presentation of our work. In accordance with your suggestion, we have revised the introduction to include a more detailed discussion of the novel aspects of our study. We have specifically emphasized the following points:

1. The novel algorithm(LGBM) we have introduced within our research framework.

2. The advancements our study contributes to the current state of knowledge and its potential to fill gaps in the existing literature.We have added the following contents to the introduction:

When developing a radiomics prediction model, the selection of an appropriate machine learning algorithm can significantly enhance the model's predictive accuracy and stability. Various classifiers, including Support Vector Machine (SVM), Logistic Regression (LR), and Random Forest (RF), have been utilized to construct models in the studies mentioned earlier. The Light Gradient Boosting Machine (LGBM), a model rooted in gradient boosting decision trees (GBDT), shares principles with the XGBoost algorithm but offers several advantages, such as faster training efficiency, lower memory consumption, higher accuracy, and support for parallel learning. To the best of our knowledge, there has been scant research evaluating the efficacy of the LGBM classifier for radiomics models based on 18F-FDG PET/CT that incorporate clinico-metabolic features for differentiating between ADC and SCC in lung cancer. Therefore, the objective of this research was to create and corroborate a superior machine learning (ML) model utilizing PET/CT data to distinguish between SCC and ADC in stage III NSCLC.

**Question 4:** The structure of this paper is confusing. Especially in the main text since Materials and Methods, there are no title numbers and subtitles, making it difficult for readers to read.

**Response:** Thank you for your valuable feedback on the structure of our manuscript. We apologize for any confusion caused by the absence of clear section numbering and subtitles in the main text.In response to your concerns, we have thoroughly reviewed the manuscript and implemented a more reader-friendly structure. We have added title numbers and subtitles to each section, and made some changes to the manuscript to improve navigation and readability throughout the paper. The revised structure is as follows:

Introduction

Materials and Methods

[Study design]

[Patients]

[18F-FDG PET/CT image acquisition ]

[Tumor Segmentation]

[Feature extraction]

[Feature selection and prediction model establishment]

[Development and validation of individualized nomogram]

[ Statistical analysis]

Results

[Clinical characteristics of patients]

[Features selection and prediction model establishment]

[Prediction performance and clinical utility of prediction models]

Discussion

Conclusions

We believe that this revised structure will greatly facilitate the reader's understanding of our research process and findings. Each section and subsection is now clearly delineated, enabling readers to quickly locate specific information within the manuscript.

**Question 5:** Are the Rad signature and Clinical signature mean the prediction values based on radiomic features and clinical features respectively? Does Rad refer to Radiomic? If so, please explain in the article or use Radiomic instead of Rad.
**Response:**Thank you for your question and for pointing out the potential ambiguity regarding the terminology used in our manuscript. We appreciate the opportunity to clarify this aspect of our work.To address your query, yes, "Rad signature" indeed refers to the predictive values based on radiomic features, and "Clinical signature" pertains to the predictive values derived from clinical features. We acknowledge that the abbreviation "Rad" may not have been immediately clear to all readers, and we apologize for any confusion this may have caused. To substantially improve readers’ comprehension of our research methods and results, we have reorganized the manuscript by discarding the subheadings associated with the Rad signature and Clinical signatures and by consolidating the corresponding paragraphs to enhance the narrative flow and coherence.

**Question 6:** The selection of features is crucial to the machine learning algorithm. It is recommended that the selected handcrafted features extracted from CT and PET images be attached and elaborated in detail.

**Response:** Thank you for your insightful comments regarding the selection of features in our machine learning algorithm. We agree that a detailed description of the selected handcrafted features is essential for the transparency and reproducibility of our study.

In response to your recommendation, we have added an additional section to the manuscript that provides a comprehensive list and description of the handcrafted features extracted from the PET/CT images. This new section can be found under the heading "3.2 Features selection and prediction model establishment" and "4 Discussion"and includes the following information:

6.1. A list of the handcrafted radiomic features selected for our predictive models.

Table 4:The final selected PET-CT radiomics features for RS model and combined model.

| Model |  | Filter |  | Feature class |  | Feature |
| --- | --- | --- | --- | --- | --- | --- |
| RS model |  | log_sigma_5_0_mm_3D |  | firstorder |  | 90Percentile |
|  |  | log_sigma_2_0_mm_3D |  | firstorder |  | Maximum.1 |
|  |  | lbp_3D_m1 |  | firstorder |  | Skewness |
|  |  | squareroot |  | ngtdm |  | Busyness |
|  |  | original |  | shape |  | Sphericity |
|  |  | wavelet_LLH |  | gldm |  | Large Dependence High Gray Level Emphasis.1 |
|  |  | wavelet_HHL |  | glszm |  | Small Area Emphasis.1 |
|  |  | exponential |  | glszm |  | Size Zone Non Uniformity Normalized |
| combined model |  | log_sigma_5_0_mm_3D |  | firstorder |  | 90Percentile |
|  |  | log_sigma_2_0_mm_3D |  | firstorder |  | Maximum.1 |
|  |  | lbp_3D_m1 |  | firstorder |  | Skewness |
|  |  | squareroot |  | ngtdm |  | Busyness |
|  |  | wavelet_LLH |  | gldm |  | Large Dependence High Gray Level Emphasis.1 |
|  |  | original |  | shape |  | Sphericity |
|  |  | logarithm |  | gldm |  | Dependence Variance.1 |

6.2. A discussion on the relevance of several important selected features to the prediction of histological subtypes, was supported by references to existing literature where applicable. The detailed information is as follows:

In our study, the most significant radiomic features for both the RS model and the combined model were lbp_3D_m1_firstorder_Skewness, log_sigma_5_0_mm_3D _firstorder_90Percentile, squareroot_ngtdm_Busyness and log_sigma_2_0_mm_ 3D_firstorder_Maximum. A previous study [29] demonstrated that first-order features were particularly stable and robust in rectal cancer. In our research, three first-order features were also confirmed to be the most significant indicators for classifying histological types. Another noteworthy feature in our study was 'busyness,' which is associated with the spatial frequency of intensity changes. Erol M et al. [30] reported that radiomic features, including busyness, were independently correlated with the staging of lung squamous cancer. Bashir et al. [13] and Hyun et al. [16] have previously explored the application of radiomics in the classification of NSCLC. However,in their studies, the radiomic features exhibiting the highest performance include separately GLSZMSZLIE, coefficient of variation, NGTDM coarseness and gray-level zone length nonuniformity, gray-level nonuniformity for zone. The best-performing subset of radiological features in our study differs from those identified in other studies. This discrepancy may be attributed to the fact that there are hundreds of radiomic features, many of which are inter-correlated, leading to the possibility that different high-ranking features might essentially represent variations of the same underlying feature.

We hope that this additional information addresses your concerns and provides a clearer understanding of how the feature selection process contributes to the performance of our machine learning algorithm.

Dear Reviewer #2:

Thank you for your candid feedback on our manuscript. We appreciate that your comments have enabled us to prepare a greatly improved manuscript.

We apologize for the previous poor formatting and some disputed descriptions of our manuscript, which may have made some of this evidence unclear.For this reason, we have revised the statements in our manuscript to better highlight the novelty and advancement of our work.

**Question 1:** Like in the manuscript abstract section, starting from purpose then conclusion which is not clear , you have to make it in a clearly understandable way.

**Response:** We thank the reviewer for pointing this out. We take your concerns seriously and are dedicated to resolving the points you’ve raised, particularly regarding the clarity of our abstract. Our revision efforts have been directed at refining the abstract to articulate our research objectives and conclusions with greater precision and coherence. By excising extraneous content and honing in on the essential results and importance of our work, we’ve crafted a more succinct abstract. Additionally, we’ve eliminated all subheadings to comply with the conventional format and to enhance the narrative flow, ensuring the abstract reads as a unified, integrated paragraph. We trust these amendments satisfactorily address your feedback, and we remain receptive to any additional input you might offer. Please find the updated abstract attached below:

Noninvasive differentiation between the squamous cell carcinoma (SCC) and adenocarcinoma (ADC) subtypes of non-small cell lung cancer (NSCLC) could benefit patients who are unsuitable for invasive diagnostic procedures. Therefore, this study evaluates the predictive performance of a PET/CT-based radiomics model. It aims to distinguish between the histological subtypes of lung adenocarcinoma and squamous cell carcinoma, employing four different machine learning techniques. A total of 255 Non-Small Cell Lung Cancer (NSCLC) patients were retrospectively analyzed and randomly divided into the training (n = 177) and validation (n = 78) sets, respectively. Radiomics features were extracted, and the Least Absolute Shrinkage and Selection Operator (LASSO) method was employed for feature selection. Subsequently, models were constructed using four distinct machine learning techniques, with the top-performing algorithm determined by evaluating metrics such as accuracy, sensitivity, specificity, and the area under the curve (AUC). The efficacy of the various models was appraised and compared using the DeLong test. A nomogram was developed based on the model with the best predictive efficiency and clinical utility, and it was validated using calibration curves. Results indicated that the logistic regression classifier had the best predictive power in the validation cohort of the radiomic model. The combined model (AUC 0.870) exhibited superior predictive power compared to the clinical model (AUC 0.848) and the radiomics model (AUC 0.774) . In this study, we discovered that the combined model, refined by the logistic regression classifier, exhibited the most effective performance in classifying the histological subtypes of NSCLC.

**Question 2:Y**ou have mentioned about "1,834 handcrafted CT features and 2,016 handcrafted PET features" , since your model is AI based what is the rational to choose this? How many from each data? How can you make it in a stantard format from each data?

**Response:** Thank you for your insightful questions regarding the selection of handcrafted features from CT and PET imaging data for our AI-based model. Here is the rationale and methodology behind our choices:

2.1. Rationale for Feature Selection:

The choice of 1,834 handcrafted CT features and 2,016 handcrafted PET features was guided by previous literature and domain expertise that suggest these features capture essential characteristics related to the pathology we are studying. These features include textural, shape, and intensity-based descriptors that have been shown to correlate with clinical outcomes. The AI model leverages these features to uncover complex patterns that may not be discernible through manual analysis or traditional statistical methods.

2.2. Distribution of Features:

From the CT data, we extracted 1,834 features, which were categorized into seven distinct groups: first-order features, shape-based features, Gray Level Dependence Matrix (GLDM) features, Gray Level Run Length Matrix (GLRLM) features, Gray Level Size Zone Matrix (GLSZM) features, Neighbouring Gray Tone Difference Matrix (NGTDM) features, and Gray Level Co-occurrence Matrix (GLCM) features. Similarly, from the PET data, we extracted 2,016 features. The number and ratio of handcrafted features were shown in Fig.2.These comprehensive feature sets allow our AI model to learn from a wide range of data characteristics, thereby enhancing its predictive performance.

2.3. Standardization of Data:

To ensure a standard format across all data, we employed a rigorous preprocessing pipeline. This involved normalization, resampling, segmentation, etc. Each feature was then z-score normalized to have a mean of zero and a standard deviation of one. This standardization process ensures that the scale of the features does not bias the AI model and allows the model to learn more effectively from both CT and PET datasets.

We believe that the combination of these handcrafted features with our AI model provides a robust framework for capturing the most relevant information from the imaging data, which is critical for accurate diagnosis and prognosis.

**Question 3:**"Lung cancer has a variety of types.Example from introduction: see this sentense : " . This is kind of incomplete sentense, you have to correct almost every sentenses.Anyway, these mentionsd are not the solid reasons for my remark , throughout the draft need a rebuild. Figures and tables are not in concise form.
**Response:**Thank you very much for your comments. We will review the entire manuscript to correct incomplete or unclear sentences, as you have indicated in your example from the introduction. Additionally, we will restructure our figures and tables to present them in a more concise and informative manner. Due to the inclusion of an additional table and a figure, we have adjusted the sequence of the tables and figures. Furthermore, with the incorporation of various machine learning methods, The figure will be simplified to display only the model constructed using the optimal machine learning method, which is the Logistic Regression algorithm.

**Fig 4 Comparison of receiver operating characteristic (ROC) curves for predicting subtype of pathology in the training and validation cohorts.**

**A shows the ROC curve of LR in the training cohort;**

**B shows the ROC curve of LR in the validation cohort.**

**Fig 5 Clinical utility of prediction models**

**A shows Nomogram of a clinical radiomics model developed based on a logistic regression model for the training cohort. gender 1:male 2:female.**

**B,C show Decision curve analysis (DCA) was conducted on the prediction model based on the logistic regression model in the training (B) and validation cohorts (C).**

**D,E show Calibration curves of the nomogram based on the logistic regression model in the training (D) and validation cohorts (E).**

We understand the magnitude of the revisions required and are prepared to undertake a comprehensive rebuild of the draft to meet the journal's standards. We assure you that we will approach these revisions with the utmost diligence and attention to detail.Once again, we are grateful for your feedback, which will undoubtedly contribute to the improvement of our work. We look forward to submitting a significantly revised manuscript that will hopefully meet your expectations and those of the journal.

Reviewer #3:

On behalf of my co-authors, we thanks very much for giving us the second opportunity to revise our manuscript. We deeply appreciate editors and reviewers for their positive and constructive comments. We would like to make an explanation to reviewer 3 and hope it could be accepted.

**Question 1:** The abstract of this manuscript should be refined.

**Response:**Thank you for your valuable comments. In response, we have carefully revised the abstract to improve both its linguistic quality and structural coherence.

Noninvasive differentiation between the squamous cell carcinoma (SCC) and adenocarcinoma (ADC) subtypes of non-small cell lung cancer (NSCLC) could benefit patients who are unsuitable for invasive diagnostic procedures. Therefore, this study evaluates the predictive performance of a PET/CT-based radiomics model. It aims to distinguish between the histological subtypes of lung adenocarcinoma and squamous cell carcinoma, employing four different machine learning techniques. A total of 255 Non-Small Cell Lung Cancer (NSCLC) patients were retrospectively analyzed and randomly divided into the training (n = 177) and validation (n = 78) sets, respectively. Radiomics features were extracted, and the Least Absolute Shrinkage and Selection Operator (LASSO) method was employed for feature selection. Subsequently, models were constructed using four distinct machine learning techniques, with the top-performing algorithm determined by evaluating metrics such as accuracy, sensitivity, specificity, and the area under the curve (AUC). The efficacy of the various models was appraised and compared using the DeLong test. A nomogram was developed based on the model with the best predictive efficiency and clinical utility, and it was validated using calibration curves. Results indicated that the logistic regression classifier had the best predictive power in the validation cohort of the radiomic model. The combined model (AUC 0.870) exhibited superior predictive power compared to the clinical model (AUC 0.848) and the radiomics model (AUC 0.774) . In this study, we discovered that the combined model, refined by the logistic regression classifier, exhibited the most effective performance in classifying the histological subtypes of NSCLC.

**Question 2:** Why the authors adopt machine learning methods for research should be clearly explained in the manuscript.

**Response:** Thank you for your comment regarding the justification for our choice of machine learning methods in our research. In response to your feedback, we will revise our manuscript to include a comprehensive explanation for adopting machine learning approaches. We will address the following contents:

As medical and information technologies advance, there is an exponential growth in the volume of medical data, especially in the production of medical imaging. These images harbor extensive latent details pertinent to human health. Yet, the manual examination and interpretation of such data are not only time-consuming but also prone to human bias. Leveraging the capabilities of machine learning can significantly alleviate these issues by extracting sophisticated features and minimizing subjectivity. Radiomics entails the quantitative retrieval of characteristics from conventional medical imaging. The development of predictive or diagnostic models through machine learning techniques allows for the collection of data that can be assimilated into clinical decision-making tools, consequently improving the accuracy of diagnoses or prognosis.[10,11].

**Question 3:** A technical roadmap for this study is suggested to be added in this manuscript.

**Response:**Thank you for your constructive suggestion to include a technical roadmap in our manuscript. We agree that a roadmap would provide readers with a clear overview of the study’s progression and the methodological steps taken.In response to your recommendation, we have added a technical roadmap as a new figure [in section :2.1 Study design, e.g. Figure 1].

**Fig. 1 The Workflow of this study.**

**Question 4:** The principle of the machine learning method used in this manuscript should be introduced in detail.

**Response:**Thank you for your valuable comment requesting a more detailed introduction to the principles of the machine learning method utilized in our study. In accordance with your suggestion, we will revise our manuscript to include a detailed section on the machine learning method we adopted. This new section can be found under the heading "4 Discussion" and includes the following information:

In the construction of radiomics-based prediction models, the selection of suitable machine learning algorithms can enhance the predictive accuracy and stability of the model. Recent advancements in machine learning algorithms, including Gaussian processes, decision trees, RF, SVM, LGBM, and LR, have propelled the application and development of radiomics. Shen [36] evaluated seven different classifiers to optimize a model for classification: SVM with a linear kernel, SVM with a radial basis function kernel (SVM-RBF), RF, LR, Gaussian process classifier (GP), linear discriminant analysis (LDA), and the AdaBoost classifier. The study found that a PET/CT radiomics model using the SVM-RBF classifier demonstrated the best performance, with an AUC of 0.9155, when integrating subregion imaging from PET-CT scans and clinical features for classifying histological subtypes of NSCLC. Parmar et al. [37] demonstrated that the random forest method was the most effective in managing radiomic feature instability, outperforming 12 other machine learning classifiers, including bagging, Bayesian, boosting, decision trees, discriminant analysis, generalized linear models, multiple adaptive regression splines, nearest neighbors, neural networks, partial least squares and principal component regression, and SVMs—in terms of prognostic performance. Huang, et al.[38] employed the LGBM algorithm to develop both a radiomic model and a fusion model (clinical + radiomic) to predict EGFR mutation status in patients with NSCLC. Models based on radiomic signatures can provide relatively accurate non-invasive predictions of EGFR expression status.

In our study, we found that the combined model constructed using the Logistic Regression algorithm performs excellently in identifying pathological subtypes, even when compared with multiple algorithms, including Light Gradient Boosting Machine. Moreover, LGBM also demonstrated good predictive performance. Logistic regression (LR), a linear model, is widely favored for binary classification problems due to its computational simplicity and interpretability. LR is adept at handling large datasets and excels with linearly separable problems. Our finding aligns with a similar study conducted by Ren et al. [35], which reported that a combined model incorporating clinico-biological features and 18F-FDG PET/CT data, utilizing the LR algorithm, showed strong capability in distinguishing SCC from ADC. Additionally, another study suggested that a model trained on 18F-FDG PET radiomics with the LR algorithm could be effective for predicting the histological subtypes of lung cancer [16].
**Question 5:** The comparison analysis between the proposed method and the existing methods is expected in this manuscript.

**Response:**Thank you for your constructive feedback regarding the need for a comparative analysis between our proposed method and existing methods in the field. We agree that such an analysis is essential to demonstrate the efficacy and novelty of our approach and to provide a clear understanding of how it stands in relation to current methodologies.To address your insightful suggestion, we will revise our manuscript to include a comprehensive comparison of our proposed method with established methods that are commonly used in similar studies.This comparative analysis will encompass the following elements, which are detailed under the heading ‘3.3 Prediction Performance and Clinical Utility of Prediction Models’ in Table 5:

Table 5 displays a consolidated overview of the predictive capabilities for differentiating ADC from SCC across multiple machine learning classifiers within the training and internal validation groups. In the validation cohorts, the LR models demonstrated superior outcomes with respect to the AUC, ACC, SEN, and SPE compared to other ML classifiers. As a result, LR was selected as the preferred ML algorithms for the classification of the specified pathological types.

| **ML** | **Data set** | **RS model** | | | | |
| --- | --- | --- | --- | --- | --- | --- |
|  |  | **AUC** | **95% CI** | **ACC** | **SEN** | **SPE** |
| **LR** | **Training** | **0.806** | **0.7432 - 0.8681** | **0.719** | **0.771** | **0.659** |
|  | **Validation** | **0.774** | **0.6692 - 0.8789** | **0.727** | **0.694** | **0.786** |
| **RF** | **Training** | **1.000** | **1.0000 - 1.0000** | **1.000** | **1.000** | **1.000** |
|  | **Validation** | **0.743** | **0.6315 - 0.8539** | **0.675** | **0.633** | **0.750** |
| **SVM** | **Training** | **0.854** | **0.7973 - 0.9106** | **0.787** | **0.792** | **0.780** |
|  | **Validation** | **0.706** | **0.5869 - 0.8242** | **0.649** | **0.510** | **0.926** |
| **LGBM** | **Training** | **0.880** | **0.8317 - 0.9274** | **0.815** | **0.979** | **0.622** |
|  | **Validation** | **0.726** | **0.6109 - 0.8417** | **0.688** | **0.592** | **0.857** |

**Table 5 Performance of four machine learning algorithms for differentiating pathological subtypes.**
**Question 6:** More recent works about machine learning should be included in this manuscript, like “A novel machine learning method for multiaxial fatigue life prediction: Improved adaptive neuro-fuzzy inference system. International Journal of Fatigue, 2024, 178: 108007.”

**Response:**Thank you for your constructive comments and for bringing the recent publication titled “A novel machine learning method for multiaxial fatigue life prediction: Improved adaptive neuro-fuzzy inference system. International Journal of Fatigue, 2024, 178: 108007” to our attention.

We agree that including more contemporary studies on machine learning will enhance the manuscript and provide a more comprehensive overview of the current advancements in the field. Accordingly, we have reviewed the suggested work. We have now incorporated a discussion of this method into the revised manuscript.We believe that this addition, along with other recent literature we have now included, will offer readers a more up-to-date perspective and underscore the significance of our study within the context of the latest developments in the field.We have added the following contents under the heading ‘4 Discussion’:

Given that different machine learning algorithms have their respective optimal application contexts, it is imperative to explore a variety of algorithms to identify the most suitable model for predicting NSCLC histology subtypes. For instance, Gao et al. proposed an improved adaptive neuro-fuzzy inference system-based machine learning method to predict the multi-axis fatigue life of various metal materials. The study found that this model exhibited superior predictive performance and extrapolation capabilities when compared with six classical machine learning models [39]. Moreover, a recent study indicated that a 3D convolutional neural network (CNN) model effectively differentiated between benign and malignant pulmonary nodules in 2-[18F]FDG PET images [40]. The optimization of machine learning algorithms should be prioritized in future research to improve the performance of predictions.

**Question 7:** In this manuscript, the features selection and prediction model establishment should be described in a more detailed approach.

**Response:**Thank you for your insightful comments regarding the need for a more detailed description of feature selection and the establishment of the prediction model in our manuscript. We appreciate your guidance in helping us enhance the clarity and depth of our work. In response to your feedback, we have revised the relevant sections of the manuscript to provide a comprehensive explanation of our methods:

To ensure maximal representation of features while maintaining their distinctiveness, we assessed the correlation among highly repeatable attributes using Spearman's rank correlation coefficient. Features exhibiting a correlation coefficient greater than 0.9 with any other feature were retained. For feature selection, we employed a greedy recursive elimination technique, which systematically removes the most redundant features from the current set at each step. Then, we employed the Least Absolute Shrinkage and Selection Operator (LASSO) regression analysis to select effective features within the training dataset. The LASSO model is particularly effective in reducing regression coefficients toward zero, thereby excluding irrelevant features by setting their coefficients to zero. The initial step involved identifying the optimal regularization parameter, λ. To accomplish this, we utilized 10-fold cross-validation, adhering to the least absolute criterion. By selecting the λ value that minimized the cross-validation error, we identified features with non-zero coefficients that were instrumental in fitting the regression model. These features were then aggregated to construct the radiomic model. Additionally, a radiomics score was computed for each patient by linearly combining the selected features with weights derived from their respective coefficients in the model. The LASSO regression modeling was conducted using the scikit-learn package in Python.

To differentiate between SCC and ADC, three independent predictive models were separately developed: the Clinical-Metabolic Model (clinic model), the PET/CT Radiomic Model (RS model), and the Combined PET/CT Radiomic and Clinical-Metabolic Model (combined model). Four machine learning classifiers, including LR, LGBM, SVM, RF ,were used to construct these models. During this process, 5-fold cross-validation was employed to derive these final models.

We would like to thank the referee again for taking the time to review our manuscript.

Sincerely,
Yalin Zhang
Clinical Research and Cultivation center of radiotherapy in Xinjiang Uygur Autonomous Region, Urumqi 830011, P.R. China
E-mail: yalin828@126.com
